# Effect of Breed on Fatty Acid Composition of Meat and Subcutaneous Adipose Tissue of Light Lambs

**DOI:** 10.3390/ani10030535

**Published:** 2020-03-23

**Authors:** Katarina Budimir, Massimo Mozzon, Marco Toderi, Paride D’Ottavio, Maria Federica Trombetta

**Affiliations:** Dipartimento di Scienze Agrarie, Alimentari ed Ambientali, Università Politecnica delle Marche, Via Brecce Bianche 10, 60131 Ancona, Italy; katarina.budimir@hotmail.com (K.B.); m.toderi@staff.univpm.it (M.T.); p.dottavio@staff.univpm.it (P.D.)

**Keywords:** fatty acids, light lamb, breed, Bergamasca, Italian Merino, Sopravissana

## Abstract

**Simple Summary:**

The recognition of the role of food in the improvement and preservation of health is receiving more and more attention among consumers, especially in developed countries. Fats from red meats are considered quite unhealthy because of their high levels of cholesterol and saturated fatty acids. Healthier lipid profiles of red meats can be achieved by a proper feed composition of animals, but other factors, such as breed, sex, and live weight, are able to affect the nutritional properties of meats. This investigation aimed to evaluate the effect of breed (Bergamasca, Italian Merino, and Sopravissana) on the fatty acid composition of invisible (intramuscular) and visible (subcutaneous) fats of light lambs reared in Central Italy transhumant farms. They recently gained a Protected Geographical Indication label (European Union, Commission Implementing Regulation No. 475/2013), as ”Agnello del Centro Italia”. It is an income opportunity for local farms that passes through the nutritional valorization of lamb meat. The indices of nutritional quality of fats have showed that the meat of Italian Merino and Sopravissana lambs had better nutritional quality than the Bergamasca breed.

**Abstract:**

Lamb meat is the main product of Central Italy transhumant farms, where lambs are traditionally reared with their mothers on pastures and are supplemented with concentrates and/or hay from day 20–30 until slaughter. However, few data are available on the fatty acid (FA) composition of unweaned lambs reared by extensive systems in Central Italy. The study aimed to evaluate the effect of breed (Bergamasca, Italian Merino, and Sopravissana) on the FA composition of intramuscular (longissimus lumborum, LL) and subcutaneous (SC) fats of light lambs. Statistical analysis showed that breed had effect only on some FAs in LL muscle fat (C18:0, C20:0, C14:1, C16:1, C17:1, C18:3 n-3, trans and conjugated linoleic acid isomers) and in SC adipose tissue (C21:0, C16:1, C18:1, C20:4 n-6, C20:5 n-3, C18:1 trans isomers). Gas chromatography data in combination with a chemometric approach could have some potential to discriminate among breeds. Indices of nutritional quality of the lipids suggested that the meat of Italian Merino and Sopravissana lambs might have better nutritional quality than Bergamasca; further studies, involving a greater number of animals, are needed to confirm these early results.

## 1. Introduction

In Mediterranean countries, consumers prefer light lambs [1], giving importance not only to the quantity but also to the quality of fat, which is defined by the levels and proportions of fatty acids (FAs). FA composition in lamb tissues can be affected by several factors, such as breed, sex, live weight, environment, degree of fatness, and interaction between these factors [2,3,4]. Moreover, some trans-FAs, which can be found in the milk and meat fat of the ruminants as a result of rumen bacterial biohydrogenation, are gaining increased attention because they are nutritionally valuable FAs [5,6,7], such as conjugated linoleic acid (CLA).

The feeding system has been shown to be one of the main factors influencing the FA composition of lamb [8], since the use of grass [2,9] or different supplements rich in polyunsaturated fatty acids (PUFA) [10] can modify the relative content of CLA and n-3 PUFA [11,12,13]. Moreover, grass feeding and grazing can increase the concentrations of CLA, n-3 PUFA, and monounsaturated fatty acids (MUFA) in lamb meat [9,14,15]. According to Juárez et al. [16], the production system (i.e., the combination of breed and diet) is the main factor to explain variations in the FA composition of light lambs. It is also known that the FA composition varies between different lamb fat depots [17], especially between intramuscular and subcutaneous fat, since each breed has specific genetic characteristics and production systems [3,18,19]. 

In Central Italy, lamb meat is the main product of transhumant farms, where lambs are reared with their mothers on pastures and are supplemented with concentrate and/or hay from day 20–30 until slaughter. Lambs are mainly produced as light lambs (carcass weight up to 13 kg) or heavy lambs (carcass weight greater than 13 kg) for Easter and Christmas markets. However, few data are available on the FA composition of unweaned lambs reared by traditional extensive systems in Central Italy [20], and little information is available on the FA composition of Bergamasca, Italian Merino and Sopravissana light lambs reared in transhumant system.

The Bergamasca is a local breed raised for lamb meat production with transhumant management. The Italian Merino breed was officially established in 1989; this sheep breed is traditionally reared in Central Appennine for meat production. The Sopravissana is an autochthonous sheep breed native of the Marche region; it is a crossbreed of local ewes and Spanish and Rambouillet rams [21,22,23].

The aim of this study was to evaluate the effect of breed (Bergamasca, Italian Merino, and Sopravissana) on the FA composition of intramuscular (IM) fat of the longissimus lumborum (LL) muscle and of the subcutaneous (SC) adipose tissue of light lambs.

## 2. Materials and Methods

### 2.1. Experimental Design, Diet, and Animal Management

The experiment started in February with 33 male single born lambs of three Italian breeds (Bergamasca, B × B; Italian Merino, IM × IM; Sopravissana, S × S; 11 lambs per breed), randomly chosen and homogeneous for birth weight (Bergamasca, kg 6.23 ± 0.99; Italian Merino, kg 5.49 ± 0.89; Sopravissana, kg 5.89 ± 0.82) (Table S1), and bred with a transhumant system in the Marche region. After lambing, all lambs were gathered with their dams that grazed on an alfalfa-dominated pasture. Dams had also free access to first cut hay and were supplemented by corn grain (0.5 kg head^−1^ day^−1^), and lambs, after 20 d since birth, received a concentrate (50% corn, 50% barley) in creep feeders ad libitum. All animals had free access to water. The average feed chemical composition analyzed according to Martillotti et al. [24], is reported in Table 1.

### 2.2. Slaughter and Sampling

The pre-slaughtering procedures were carried out in compliance with standards reported in the Annex 3 of the Council Regulation (EC) No. 1099/2009. The 33 lambs were slaughtered in a commercial slaughterhouse at the average age of 60 d, recording the following live weight: Bergamasca, kg 23.35 ± 4.44; Italian Merino, kg 24.70 ± 5.29; and Sopravissana, kg 25.21 ± 2.38 (Table S1). At 24 h postmortem, samples of the longissimus lumborum muscle and adipose tissue were taken from the left side of the carcasses, between the 1st and 6th lumbar vertebrae. The samples were then frozen at −20 °C until the analytical determinations.

### 2.3. Determination of Total Fatty Acid Composition

Total lipids from the freeze-dried muscle samples and from the adipose tissue were extracted by Soxtec^TM^ (FOSS Italia s.p.a., Padova, Italy) system with the use of petroleum ether [24]. The preparation of fatty acids methyl esters (FAMEs) and their gas chromatographic analysis were carried out according to Haddad et al. [25,26]. The FAME composition of LL and SC were determined by a gas chromatograph HRGC MEGA 2 (Fisons Instruments, Milano, Italy) equipped with a Rt-2560® column (0.25 mm × 100 m, 0.20 µm film thickness, Restek, PA, USA). FAMEs were quantified (g kg^−1^ of total FAs) by the peak area normalization method.

To assess the nutritional quality of the intramuscular and subcutaneous fats, the PUFA/SFA and the PUFA n-6/n-3 ratios were calculated. Moreover, the Atherogenic Index (AI) and Thrombogenic Index (TI) were calculated according to the formulas used by Trombetta et al. [27], while the hypocholesterolemic/Hypercholesterolemic ratio (h/H) was calculated according to Fernández et al. [28].

### 2.4. Data Analysis

Statistical analyses were conducted with the software JMP® Version 10 (SAS Institute Inc., Cary, NC) [29], using a one-way analysis of variance (ANOVA) to evaluate the breed effect for all variables (IM and SC fatty acids and nutritional indices). The data of intramuscular and subcutaneous fat were analyzed separately. Multiple comparisons among means were carried out through the Tukey-Kramer’s Honest Significant Difference (HSD) test and the level of significance was set to 0.05. Measured variables (FAs), whose mean value was above 1 g kg^−1^ (of total FAs) and the nutritional indices were included in the multivariate analysis. Variable reduction was achieved by Principal Component Analysis (PCA) on variance-covariance matrix. Mean centered data were used to optimally describe the orientation of scores and loadings. 

## 3. Results

### 3.1. ANOVA Analysis

No significant differences among groups (breeds) were observed in the lamb weights at slaughtering. Total FA compositions of LL muscle and SC adipose tissue of Bergamasca, Italian Merino, and Sopravissana lambs are reported in Table 2. Ratios and indices useful to evaluate the nutritional quality of LL muscle and SC adipose tissue are presented in Table 3.

#### 3.1.1. Saturated Fatty Acids

Bergamasca lambs showed for significantly higher values of capric acid (C10:0) and stearic acid (C18:0) (6.5 ± 1.1 g kg^−1^ and 159.0 ± 17.5 g kg^−1^, respectively) than Italian Merino (4.9 ± 1.2 g kg^−1^ and 118.9 ± 18.5 g kg^−1^) and Sopravissana (5.4 ± 0.9 g kg^−1^ and 114.7± 24.5 g kg^−1^) in LL muscle. Higher content of arachidic acid (C20:0) in LL muscle was also detected in Bergamasca lambs, compared to the other breeds (1.4 vs. 0.8 g kg^−1^). Because of significantly higher values of previously mentioned FAs, Bergamasca lambs had the highest amount of total SFA in LL muscle compared to the other two breeds (539.0 vs. 488.7 and 504.7 g kg^−1^). Regarding SC adipose tissue, C21:0 was the only SFA affected by breed, with Bergamasca lambs having higher values than Italian Merino and Sopravissana ones (2.6 vs. 0.8 and 1.0 g kg^−1^).

#### 3.1.2. Unsaturated Fatty Acids

The analysis of the MUFA profile showed for Bergamasca lambs, compared to the other two breeds, a significantly lower percentage of palmitoleic acid (C16:1) in LL muscle (14.6 vs. 20.1 and 20.5 g kg^−1^) and in SC adipose tissue (10.6 vs. 12.6 and 13.8 g kg^−1^). A lower content of C14:1 (2.2 vs. 3.2 and 3.6 g kg^−1^) and C17:1 (4.7 vs. 6.1 and 6.2 g kg^−1^) in LL muscle was also observed in Bergamasca lambs than in Italian Merino and Sopravissana ones. The major MUFA was ∑C18:1c (sum of oleic and cis-vaccenic acids), both in the LL muscle (276.9 g kg^−1^) and SC adipose tissue (282.7 g kg^−1^), and was significantly lower in Bergamasca lambs. Trans isomers of C18:1 were significantly higher in the SC fat of Bergamasca (49.0 vs. 13.1 and 29.2 g kg^−1^) than of Italian Merino and Sopravissana. Total MUFA content in SC adipose tissue from Bergamasca (299.4 g kg^−1^) was significantly lower than in Italian Merino lambs (347.6 g kg^−1^).

Some PUFAs were also affected by breed. Italian Merino and Sopravissana lambs showed significantly higher values of C18:2 cis-9, trans-11 (CLA) and ∑C18:2t in LL muscle. Moreover, α-linolenic acid (C18:3 n-3) in Bergamasca LL muscle was significantly lower than Italian Merino (14.0 vs. 16.3 g kg^−1^), while no differences between breeds were observed for this fatty acid in SC adipose tissue. The arachidonic acid (C20:4 n-6) value of SC fat was significantly influenced by breed, showing higher value in Bergamasca lambs than in other breeds (2.2 vs. 0.4 and 0.7 g kg^−1^). Moreover, Bergamasca lambs had a higher value of eicosapentaenoic acid (EPA; C20:5 n-3) in SC adipose tissue than Italian Merino (0.6 vs. 0.2 g kg^−1^), while other long chain n-3 PUFAs (DPA, C22:5 n-3, and DHA, C22:6 n-3) were not affected by breed, both in LL muscle and SC adipose tissue.

The Bergamasca breed showed a significantly lower content of total UFA in LL muscle (377.6 vs. 415.0 g kg^−1^) and therefore less favorable IM fat composition than Italian Merino lambs.

#### 3.1.3. Nutritional Indices

A significant difference occurred in PUFA/SFA ratio of LL muscle (Table 3), with Bergamasca lambs having a lower ratio than Italian Merino ones (0.15 vs. 0.20, respectively). However, the PUFA/SFA ratios of the three breeds were relatively low (range: 0.15–0.20 and 0.09–0.10 in LL and SC adipose fats, respectively) and below the recommended value for human health (0.4), as suggested by Wood et al. [30].

The high proportion of PUFAs is not necessarily healthy, if the n-6/n-3 ratio is not properly balanced. Values of n-6/n-3 ratio ranged from 1.38 to 1.52 and from 1.72 to 1.83 for IM and SC fat respectively, and resulted within the nutritional recommendations for human diet, as this ratio should range between 1 and 4 [31].

The h/H ratio is used as an index of the cholesterolemic effect of the fat source, and it was not affected by breed.

The AI and TI values for all LL muscle and SC adipose tissue samples were higher than the recommended value for a healthy diet (1.0, [32]). A significantly higher TI was found for intramuscular fat from LL muscle of Bergamasca lambs than for Italian Merino and Sopravissana lambs (1.99 vs. 1.62 and 1.75). However, the fat content of Bergamasca LL muscle was significantly lower (1.55 ± 0.65%) than Italian Merino (1.97 ± 0.54%) and Sopravissana (1.92 ± 0.57%) lamb meats (Table S1).

### 3.2. Multivariate Analysis

**Figure 1:**
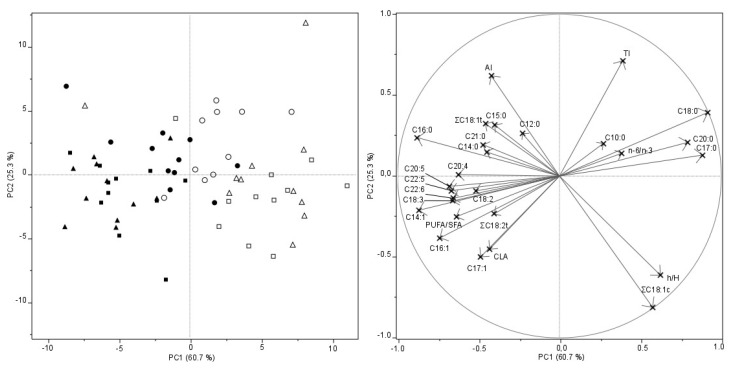
Left: PCA scores plot of intramuscular (IM) and subcutaneous (SC) fat samples. Symbols are: Bergamasca, IM; Sopravissana, IM; Italian Merino, IM; Bergamasca, SC; Sopravissana SC; Italian Merino, SC. Right: PCA loadings plot of variables (individual and ratios).

Experimental data (individual FAs and ratios) were explored by means of PCA to evaluate the structure of variables (measured and derived from measured parameters) and objects (i.e., samples). The inclusion of variable combinations (nutritional indexes) affected neither the sample distribution on the scores plot nor the relationships among variables on the loadings plot. The first two PCs globally explained 86% of the total variability of the samples in terms of their FA composition. The loadings plot in Figure 1 showed that ∑C18:1c (sum of oleic and cis-vaccenic acids) and a cluster of longer SFA (C18:0, C17:0, C20:0) had positive loadings on PC1, while shorter SFA (C12:0, C14:0, C15:0, C16:0) had negative loadings, as well as all PUFA and trans-FAs (∑C18:1t, ∑C18:2t, CLA). On the contrary, PC2 was more affected by nutritional indices (TI and AI with high positive loadings; h/H with negative loading) than by measured variables, except for ∑C18:1c. The loadings plot clearly reflected the biochemical closeness inside the n-3 (C18:3, C20:5, C22:5, C22:6) and n-6 (C18:2, C20:4) clusters. Also, the biological precursors of the families, linoleic (C18:2) and α-linolenic (C18:3) acids, were positively correlated. As expected, PUFA/SFA ratio was positively correlated with all the PUFAs cited above, but it was negatively correlated only with the SFA C18:0. A positive correlation was also found between h/H and ∑C18:1c. The strongest inverse correlations were found between the pairs AI vs. h/H, TI vs. PUFA/SFA, and AI vs. ∑C18:1c.

The scores plot in Figure 1 shows the distribution of the samples on the plane defined by PC1 and PC2. The lamb fats were well differentiated depending on the body part (LL muscle vs. SC fat): SC fat samples had positive relationships with variables having positive loadings in PC1 (mainly C18:0 and ∑C18:1c, but also with C10:0, C17:0, and C20:0), while IM fats had positive relationships with variables having negative PC1 loadings (shorter SFA, PUFA, trans-FAs, and CLA). Hence, PC1 was able to distinguish between IM and SC fats, the latter being characterized by higher values of ∑C18:1c (276.9–288.8 vs. 282.7–327.9 g kg^−1^), C18:0 (114.7–159.0 vs. 188.6–203.5 g kg^−1^), C17:0 (12.1–13.4 vs.15.0–16.4 g kg^−1^), C20:0 (0.8–1.4 vs.1.9–2.0 g kg^−1^), and lower values of C16:0 (234.6–247.9 vs. 202.3–211.8 g kg^−1^), n-6 PUFA (37.2–44.6 vs. 21.7–23.0 g kg^−1^), n-3 PUFA (23.9–30.0 vs. 12.4–12.8 g kg^−1^), and CLA (18.1–22.0 vs. 14.5–17.8 g kg^−1^). PC2 was somewhat useful for differentiating among breeds. In fact, Bergamasca samples were generally characterized by positive loadings on PC2, while Sopravissana and Italian Merino samples, which are genetically correlated, mainly lay undifferentiated on the negative PC2 space. Higher values of TI characterized SC fats of Bergamasca (2.48), while h/H values and ∑C18:1c levels “drove” the differentiation of Italian Merino and Sopravissana SC fat composition (lower right quarter) away from that of Bergamasca breed (upper right quarter). Higher AI values characterized the FAs composition of LL-IM fat of Bergamasca, while LL-IM samples of Italian Merino and Sopravissana were “pulled” towards negative scores (lower left quarter), mainly by unsaturated fatty acids (C14:1, C17:1, C16:1, n-3 PUFA, C18:2, CLA).

## 4. Discussion

### 4.1. Saturated Fatty Acids

The intramuscular FA contents of the present study were comparable to those of Italian Merino light lambs reared according to a similar production system [33], and of some other Italian sheep breeds slaughtered at similar age, such as Apulian [34], Appenninica [20], Leccese, and Comisana [35].

The SC adipose fat of breeds did not differ in total SFA content, which was similar to that of the SC fat of Grazalema Merino light lambs reported by Juárez et al. [3]. However, total SFAs obtained in the present study were higher than those reported in Mouflon × Sarda and Sarda × Sarda suckling lambs [17]. Particularly, saturated fatty acids C16:0 and C20:0 had values comparable to those reported by Vacca et al. [17] in the IM fat of suckling lambs Mouflon × Sarda and Sarda × Sarda.

### 4.2. Unsaturated Fatty Acids

The content of ∑C18:1c was similar to the values of oleic acid reported by Mazzone et al. [20] for the IM fat of LL Apennine, by della Malva et al. [36] for lambs slaughtered at 75 d since birth, and by Santos-Silva et al. [37] for lambs slaughtered at 24 kg live weight. The total MUFA content in LL muscle from all breeds (range: 298.4–318.4 g kg^−1^) was lower than detected by Mazzone et al. [20] in the LL intramuscular fat of Apennine (361.9 g kg^−1^), and in Leccese and Comisana light lambs (365.5 g kg^−1^ and 375.0 g kg^−1^), slaughtered at the same age [35].

Linoleic acid percentage (C 18:2 n-6) in LL muscle was much lower than average values reported for Apennine [20], Italian Merino [33], and Altamurana lambs [35] slaughtered at similar ages.

Values of C18:2cis-9, trans-11 were higher than those reported by Mazzone et al. [20] for the LL intramuscular fat of Apennine lambs, Leccese, and Comisana slaughtered at 60 d [35] and for Merino Branco and Ile de France × Merino Branco light lambs with similar rearing systems [37]. Again, CLA levels were higher than the average value (1.02%) reported for Sarda suckling lambs reared with their mothers at pasture and slaughtered at 30–40 days of age [38]; in fact, it is known that grass-fed lambs tend to have higher amounts of rumenic acid than concentrate-fed ones [2,9,39]. Meat from ruminants has higher levels of CLA than meat from non-ruminants, as a product of bacterial isomerization or/and biohydrogenation of PUFA in the rumen. Moreover, the CLA content of the meat is slightly modified by cooking. CLA (C18:2 cis-9, trans-11), found in the meat and milk of ruminants, has benefits for human health such as anticancerogenic, antidiabetic, and other positive effects [40].

The observed MUFA values of SC adipose tissue were lower than the SC fat of Grazalema Merino and Churra Lebrijana light lambs (40.10 and 41.38%, respectively), as reported by Juárez et al. [3].

The mean value for α-linolenic acid in LL muscle (15.1 g kg^−1^) was similar to that reported for the IM fat of Muflon × Sarda and Sarda × Sarda suckling lambs [17] and for the LL fat of Apennine lambs reared in winter [20], but higher than Merino Branco and Ile de France × Merino Branco light lambs [37], and Leccese (0.91%) and Comisana (1.0%) light lambs slaughtered at same age [35].

Santos-Silva et al. [37] and Marino et al. [41] reported higher values of EPA in the IM fat of light lambs reared with ewes on pasture and supplemented with hay and a concentrate, as well as higher values of docosahexaenoic acid (DHA; C22:6 n-3).

The PUFA mean value of the three breeds in the LL muscle (87.1 g kg^−1^) was similar to that reported for Leccese (8.91%), and lower than reported for Comisana (10.94%) light lambs [35], Altamurana lambs (17.24%) [36], Italian Merino lambs (16.09%) [33] slaughtered at a similar age (60–70 d), Grazalema Merino (15.75%), and Churra Lebrijana (14.39%) light lambs [3]. The mean value of total PUFA in SC adipose tissue (51.3 g kg^−1^) was halfway between those reported for Grazalema Merino (4.97%) and Churra Lebrijana (5.91%) light lambs [3]. Some authors reported that differences in n-3 PUFA are influenced by the feeding system and the presence/absence of dry/fresh forage [37,42].

The difference in the FA composition of the SC adipose tissue was significantly affected by breed in accordance with the study of Juarez et al. [3].

### 4.3. Nutritional Indices

The PUFA/SFA ratio of Italian Merino and Sopravissana was comparable with the ratio observed in Leccese (0.17) and Comisana (0.21) lambs slaughtered at 45 and 60 d respectively [35], while higher values are reported for Sarda suckling (0.55) [38], Apennine light (0.42) [20], and Italian Merino lambs (0.25) [33].

A higher n-6/n-3 ratio was found for purebred Sarda lambs and crossbred Mouflon × Sarda exclusively milk fed [17], as well as for light lambs reared with their dams on pasture and supplemented with hay and a concentrate [36,37,43].

The h/H values observed in the LL intramuscular fat and SC adipose tissue samples (range: 1.01–1.23) were lower than those reported for different breeds of light lambs [32,37], and could be ascribed to breed and different fat depositions, as suggested by Sinanoglou et al. [32].

The AI and TI values in LL muscle were comparable to data reported by D’Alessandro et al. [35] for Leccese lambs slaughtered at 60 d. Oriani et al. [33] reported an analogous TI value of intramuscular fat, while lower values are reported for Altamurana light lambs [36], and for lambs of different breeds [32]. Vacca et al. [17] also reported lower values of AI and TI for the IM fat of suckling lambs, while values for these indices reported for adipose tissue are comparable with the ones resulting in the present study.

## 5. Conclusions

The present study, albeit carried out on a limited number of samples, to our knowledge, is the first one reporting a comparison of detailed the FA profiles of light lambs reared according to the traditional extensive management of Central Italy.

The FA profile of the IM fat and SC tissue analyzed was characterized by high values of monounsaturated FAs (∑C18:1c) and longer SFAs (C18:0, C17:0, C20:0), CLA, and by low values of n-6 and n-3 PUFA.

Differences among breeds occurred just for some FAs, but always as differences between Bergamasca and the other two breeds, both in LL muscle and SC adipose tissue. Therefore, probably due to the similar genotype, Italian Merino, and Sopravissana (both Merino derived breeds) had a very similar FA composition. Despite the limited number of significant differences in FA composition, gas chromatography data in combination with a chemometric approach could have some potential in discriminating among breeds.

In general, the meat from Bergamasca lambs presented lower CLA as well as higher TI, compared to Italian Merino and Sopravissana. However, the meat and subcutaneous adipose tissue of all three breeds had a high content of health promoting n-3 fatty acids and CLA. The ratios between n-6/n-3 fatty acids of both LL muscle and SC adipose tissue were low and in accordance with the recommended values for human health.

## Figures and Tables

**Table 1:** Dry matter (DM, g kg^−1^) and chemical composition (g kg^−1^ of DM) of the different feeds.

Parameter	Pasture	Hay	Corn Grain	Concentrate
DM	215.1	883.1	835.2	858.9
Crude protein	155.8	73.4	56.6	76.8
Ether extract	16.5	10.5	28.8	27.3
Crude fibre	214.6	312.8	26.5	29.4
NDF	396.5	634.5	163.0	219.9
ADF	301.1	410.2	30.3	49.0
ADL	63.7	61.5	7.6	15.2

NDF, Neutral Detergent Fiber; ADF, Acid Detergent Fiber; ADF, Acid Detergent Fiber.

**Table 2:** Fatty acid composition (g kg^−1^ of total fatty acid, as methyl esters) of the total lipids of the longissimus lumborum muscle (LL) and of subcutaneous adipose tissue (SC) from light lambs (mean ± SD).

Fatty Acid	LL Intramuscular Fat			SC Adipose Tissue		
	Bergamasca (*n* = 11)	It. Merino (*n* = 11)	Sopravissana (*n* = 11)	Bergamasca (*n* = 11)	It. Merino (*n* = 11)	Sopravissana (*n* = 11)
C10:0	6.5 ^a^ ± 1.1	4.9 ^b^ ± 1.2	5.4 ^ab^ ± 0.9	8.1 ± 1.8	7.3 ± 1.8	6.7 ± 1.3
C11:0	0.4 ± 0.1	0.4 ± 0.1	0.4 ± 0.1	0.4 ± 0.1	0.4 ± 0.1	0.4 ± 0.1
C12:0	14.5 ± 2.5	13.0 ± 2.9	13.7 ± 2.1	16.8 ± 2.7	15.0 ± 4.7	13.8 ± 3.5
C13:0	0.9 ± 0.1	0.9 ± 0.2	1.0 ± 0.1	1.0 ± 0.2	1.0 ± 0.3	1.0 ± 0.2
C14:0	93.8 ± 10.9	92.0 ± 11.3	97.7 ± 8.2	100.8 ± 10.2	95.8 ± 16.9	93.8 ± 18.3
C15:0	8.7 ± 0.9	8.2 ± 1.0	8.6 ± 0.5	9.5 ± 0.8	8.5 ± 1.7	8.3 ± 1.5
C16:0	237.2 ± 1.9.7	234.6 ± 15.5	247.9 ± 14.6	211.8 ± 18.6	203.9 ± 20.4	202.3 ± 30.7
C17:0	13.4 ± 1.5	12.3 ± 1.2	12.1 ± 1.2	15.0 ± 1.3	15.7 ± 1.0	16.4 ± 1.5
C18:0	159.0 ^a^ ± 17.5	118.9 ^b^ ± 18.5	114.7 ^b^ ± 24.5	188.6 ± 30.6	196.3 ± 28.9	203.5 ± 36.3
C20:0	1.4 ^a^ ± 0.5	0.8 ^b^ ± 0.3	0.8 ^b^ ± 0.1	1.9 ± 0.4	1.9 ± 0.1	2.0 ± 0.3
C21:0	3.1 ± 0.9	2.6 ± 1.2	2.3 ± 0.6	2.6 ^a^ ± 1.1	0.8 ^b^ ± 0.5	1.0 ^b^ ± 0.5
C14:1	2.2 ^b^ ± 0.8	3.2 ^a^ ± 0.6	3.6 ^a^ ± 0.8	1.5 ± 0.4	1.4 ± 0.3	1.5 ± 0.4
C16:1 n-7	14.6 ^b^ ± 3.7	20.1 ^a^ ± 2.7	20.5 ^a^ ± 3.7	10.6 ^b^ ± 2.3	13.8 ^a^ ± 1.7	12.6 ^ab^ ± 2.0
C17:1	4.7 ^b^ ± 1.1	6.2 ^a^ ± 0.7	6.1 ^a^ ± 1.1	4.7 ± 0.6	4.5 ± 0.8	4.6 ± 0.4
∑C18:1t	38.9 ± 14.5	48.4 ± 8.3	47.2 ± 8.2	49.0 ^a^ ± 13.7	13.1 ^c^ ± 5.0	29.2 ^b^ ± 18.2
∑C18:1c	276.9 ± 30.8	288.8 ± 29.7	284.6 ± 20.0	282.7 ^b^ ± 16.9	327.9 ^a^ ± 27.2	308.2 ^ab^ ± 44.0
C18:2 cis-9, trans-11 (CLA)	18.1 ^b^ ± 2.5	22.0 ^a^ ± 3.6	21.8 ^a^ ± 4.2	14.5 ± 5.5	17.8 ± 2.9	16.5 ± 2.9
∑C18:2t	15.6 ^b^ ± 2.1	18.7 ^a^ ± 2.4	19.2 ^a^ ± 3.0	15.5 ± 1.8	16.7 ± 1.9	17.5 ± 2.6
C18:2 n-6	29.6 ± 10.8	34.9 ± 10.2	29.0 ± 10.9	19.6 ± 2.1	20.8 ± 1.9	21.6 ± 2.9
C18:3 n-3	14.0 ^b^ ± 1.9	16.3 ^a^ ± 1.9	14.9 ^ab^ ± 2.3	9.9 ± 1.1	10.7 ± 1.3	10.4 ± 1.8
C20:2 n-6	0.8 ± 0.3	0.8 ± 0.1	0.8 ± 0.1	0.8 ± 0.4	0.4 ± 0.2	0.7 ± 0.3
C20:4 n-6	6.9 ± 3.0	8.9 ± 6.5	7.4 ± 2.5	2.2 ^a^ ± 1.4	0.4 ^b^ ± 0.2	0.7 ^b^ ± 0.5
C20:5 n-3 (EPA)	3.4 ± 1.3	5.0 ± 2.9	4.0 ± 1.5	0.6 ^a^ ± 0.5	0.2 ^b^ ± 0.2	0.3 ^ab^ ± 0.2
C22:5 n-3 (DPA)	4.6 ± 1.1	6.1 ± 3.2	5.2 ± 1.5	1.5 ± 0.5	1.4 ± 0.6	1.5 ± 0.4
C22:6 n-3 (DHA)	1.9 ± 0.6	2.7 ± 1.5	2.4 ± 0.6	0.5 ± 0.2	0.4 ± 0.3	0.5 ± 0.2
Other fatty acids	28.9 ± 2.2	29.2 ± 2.8	28.8 ± 2.0	30.2 ^a^ ± 1.6	23.9 ^b^ ± 2.3	24.7 ^b^ ± 2.2
SFA	539.0 ^a^ ± 22.5	488.7 ^b^ ± 24.0	504.7 ^b^ ± 31.2	556.4 ± 32.4	546.5 ± 31.7	549.3 ± 35.5
MUFA	298.4 ± 28.3	318.4 ± 28.9	314.7 ± 22.2	299.4 ^b^ ± 19.1	347.6 ^a^ ± 28.5	327.0 ^ab^ ± 44.9
PUFA	79.2 ± 16.0	96.6 ± 24.9	85.4 ± 19.2	49.5 ± 7.2	52.2 ± 6.2	52.2 ± 6.4
UFA	377.6 ^b^ ± 23.7	415.0 ^a^ ± 28.6	400.2 ^ab^ ± 26.8	348.9 ^b^ ± 25.5	399.8 ^a^ ± 33.0	379.2 ^ab^ ± 47.1
n-6 PUFA	37.2 ± 12.9	44.6 ± 16.7	37.2 ± 12.7	22.6 ± 3.4	21.7 ± 2.1	23.0 ± 3.1
n-3 PUFA	23.9 ± 3.9	30.0 ± 9.0	26.5 ± 5.1	12.4 ± 2.0	12.8 ± 2.2	12.7 ± 2.2

Values with different letters in the same row are significantly different (*p* < 0.05). ∑C18:1t = sum of unidentified C18:1 trans isomers; ∑C18:1c = C18:1∆9c + ∆11c; ∑C18:2t = 18:2 n-6 c,t + t,c + t,t; SFA, Saturated Fatty Acids = Σ (C10:0, C11:0, C12:0, C13:0, C14:0, C15:0, C16:0, C17:0, C18:0, C20:0, C21:0); MUFA, Monounsaturated Fatty Acids = Σ (C14:1, C16:1 n-7, C17:1, Σ18:1c); PUFA, Polyunsaturated Fatty Acids = (C18:2 n-6, C18:3 n-3, C18:2 c9t11, C20:2 n-6, C20:4 n-6, C20:5 n-3, C22:5 n-3, C22:6 n-3); UFA, Unsaturated Fatty Acids = (MUFA + PUFA); n-6 PUFA= Σ (C18:2n-6, C20:2n-6, C20:4n-6); n-3 PUFA =∑ (C18:3n-3, C20:5n-3, C22:5n-3, C22:6n-3).

**Table 3:** Ratios and indices of fatty acids (as relative percentages) determined in the total lipids of the longissimus lumborum muscle (LL) and of subcutaneous adipose tissue (SC) from light lambs (mean ± SD).

Fatty Acid	LL Intramuscular Fat			SC Adipose Tissue		
	Bergamasca (*n* = 11)	It. Merino (*n* = 11)	Sopravissana (*n* = 11)	Bergamasca (*n* = 11)	It. Merino (*n* = 11)	Sopravissana (*n* = 11)
PUFA/SFA	0.15 ^b^ ± 0.03	0.20 ^a^ ± 0.06	0.17 ^ab^ ± 0.04	0.09 ± 0.02	0.10 ± 0.02	0.10 ± 0.02
n-6/n-3	1.52 ± 0.39	1.48 ± 0.15	1.38 ± 0.34	1.83 ± 0.15	1.72 ± 0.13	1.82 ± 0.21
h/H	1.03 ± 0.15	1.12 ± 0.17	1.01 ± 0.10	1.02 ± 0.11	1.23 ± 0.21	1.19 ± 0.26
AI	1.76 ± 0.30	1.58 ± 0.26	1.73 ± 0.19	1.90 ± 0.21	1.60 ± 0.36	1.68 ± 0.50
TI	1.99 ^a^ ± 0.19	1.62 ^b^ ± 0.22	1.75 ^b^ ± 0.20	2.48 ± 0.33	2.20 ± 0.34	2.33 ± 0.46

Values with different letters in the same row are significantly different (*p* < 0.05). SFA = Σ (C10:0, C11:0, C12:0, C13:0, C14:0, C15:0, C16:0, C17:0, C18:0, C20:0, C21:0); MUFA = Σ (C14:1, C16:1 n-7, C17:1, Σ18:1c); PUFA = (C18:2 n-6, C18:3 n-3, C18:2 c9t11, C20:2 n-6, C20:4 n-6, C20:5 n-3, C22:5 n-3, C22:6 n-3); UFA = (MUFA + PUFA); n-6 = Σ (C18:2n-6, C20:2n-6, C20:4n-6); n-3 =∑ (C18:3n-3, C20:5n-3, C22:5n-3, C22:6n-3); h/H, hypocholesterolemic/Hypercholesterolemic ratio = (C18:1n-9 + C18:2n-6 + C18:3n-3 + C20:2n-6 + C20:4n-6 + C20:5n-3 + C22:5n-3 + C22:6n-3)/(C14:0 + C16:0); AI, Atherogenic Index = (C12:0 + 4 × C14:0 + C16:0)/ (MUFA + n-6 PUFA + n-3 PUFA); TI, Thrombogenic Index = (C14:0 + C16:0 + C18:0)/ [0.5 × MUFA + 0.5 × n-6 PUFA + 3 × n-3 PUFA + (n-3/n-6)].

## Supplementary Materials

The following are available online at https://www.mdpi.com/2076-2615/10/3/535/s1, Table S1: Weights at birth and slaughtering and intramuscular fat content of longissimus lumborum muscle of lambs used for experiment.
